# Comparing *Aedes vigilax* Eggshell Densities in Saltmarsh and Mangrove Systems with Implications for Management

**DOI:** 10.3390/insects5040984

**Published:** 2014-12-12

**Authors:** Pat Dale, Jon Knight, Lachlan Griffin

**Affiliations:** 1 Environmental Futures Research Institute, Griffith University, Nathan Queensland 4111, Australia; 2 Australian Rivers Institute, Griffith University, Nathan Queensland 4111, Australia; E-Mail: j.knight@griffith.edu.au; 3 Griffith School of Environment, Griffith University, Nathan Queensland 4111, Australia; E-Mail: lachlan.griffin@inbox.com

**Keywords:** *Aedes vigilax*, eggshells, mosquito management, mangrove, saltmarsh

## Abstract

*Aedes vigilax* (Skuse), a nuisance and disease vector, is prolific in intertidal wetlands in Australia. Aedine mosquitoes oviposit directly onto substrate. The eggshells are relatively stable spatially and temporally, providing an estimate of mosquito larval production. The aims of the research were to compare, at a general level, oviposition in mangroves and saltmarshes, and to compare oviposition between different habitats within mangroves and saltmarshes. The results indicated that there were no significant differences between production in mangrove and saltmarsh overall. However, within each system there were significant differences between habitat classes, with mangrove hummocks being the most productive. All classes, except for fringing mangrove forests, produced sufficient densities of eggshells (>0.05/cc) to warrant concern. While mosquito production in mangroves is known, the significantly higher production rates in the mangrove hummock habitats had not been demonstrated. This warrants improved management strategies that both specifically target these parts of mangrove systems and, secondly, addresses the longer-term potential for mangrove hummock habitats developing in the future; such as, in response to sea level rise and mangrove encroachment into saltmarsh. A strategy to increase tidal flushing within the systems would improve water quality and mitigate adverse impacts while providing a source reduction outcome.

## 1. Introduction

*Aedes vigilax* (Skuse) is the major species in intertidal wetlands (saltmarsh and mangroves) and is of medical significance in much of subtropical to tropical Australia. Further, it transmits Ross River and Barmah Forest viruses to humans. Both diseases are alphaviruses with a life cycle that involves an intermediate host such as macropods (kangaroos and wallabies) and domestic animals [1]. Ross River virus has a higher incidence than Barmah Forest virus. Between 1993 and 2013, 50% of all Australian cases of Ross River virus were in the subtropical-tropical state of Queensland, ranging from 886 to 4881 cases reported each year (data from Australian Government, Department of Health 2014) [2].

Mosquito management is a major part of controlling the disease and there is evidence that it does reduce the incidence of Ross River virus [3]. In Australia, mosquito management tends to focus on larval control, as larvae are concentrated into habitats that can be treated with larvicides and/or by source reduction, thereby minimising the need for adulticiding very near to, or in areas of, human settlement. Knowing where the larval habitats are is an essential prerequisite for developing appropriate management strategies that maximise management success and minimise both environmental and economic impacts from the risk of over treatment.

The oviposition behavior of gravid females determines larval habitats and the availability of suitable environmental conditions subsequent to hatching including drying to condition eggs and flooding for hatching and survival of larvae. Tidal flooding patterns and topography are two major controls governing environmental suitability for *Ae vigilax* production with the insect’s lifecycle timed to spring-tide dynamics. Consequently, marsh surface elevation is important with higher marsh surfaces that experience less frequent tidal connection being more suitable than lower marsh elevations that experience more frequent tidal connections. *Aedes taeniorhynchus* production in Florida marshes is similarly controlled by tides and topography. These conditions are explained more fully in [4,5,6].

One way to identify larval habitats is to go back one stage in the mosquito life cycle and investigate the pattern of oviposition, on the assumption that where there are eggshells there have been larvae. Research conducted in Florida [7,8,9] related oviposition preferences of *Ae taeniorhynchus* (Wiedemann) and larval production to habitat characteristics using occurrence and density of both eggs and eggshells in red and black mangroves. The research found that: oviposition occurred independently of mangrove forest type; using either eggs or eggshells indicated oviposition; and estimation using either density or occurrence measures was an effective indicator of oviposition. Subsequently, this approach has been successfully used for other intertidal aedine mosquitoes that lay eggs on the substrate [4,10,11,12,13]. Eggshells are stable, with a surface texture that adheres to the substrate [7,14,15], they remain for considerable periods of time [16] and hence are reliable indicators of larval habitats in the vicinity. A value of >0.05 eggshells/cc indicates that a site is likely to produce significant mosquito numbers [8].

A major benefit of eggshell sampling is that it can be conducted at any time suitable for collection of sediment samples, thus can be undertaken across an entire area of concern providing a detailed and longer-term habitat use perspective. Conversely, larval survey must be timed to hatching triggers and therefore the habitat needs to be accessed when wet, usually when much of the habitat is inaccessible. The benefit of larval survey is that it provides timely information on the nature of any given hatch and this is needed for day-to-day control operations.

Most research on eggshell distribution has been done on saltmarshes but there has been some in mangroves [4,7]. None has compared the two habitats in terms of their structure or relative importance for oviposition (and hence potential larval production). Addressing this information gap has value for management and can enable resources to be focussed on the areas of maximum larval production.

The aim of this paper is to collate and compare eggshell data from both saltmarsh and mangrove sites. It addresses two questions:

1. Is oviposition at a general level different between saltmarsh and mangrove sites?
2. Is oviposition different between habitats within mangroves and saltmarshes?

## 2. Experimental

### 2.1. Study Areas

Eggshell data were collected from ten saltmarsh and five mangrove sites between Noosa, Queensland (26°23' S, 153°03' E) and Tweed Shire, New South Wales (28°13' S, 153°30' E), over a linear distance of ~200 km. In the salt marsh the vegetation was dominated by *Sporobolus virginicus* (L.) Kunth and or *Sarcocornia quinqueflora* (Bunge ex Ung.-Sternb.) In the mangroves the vegetation was dominated by *Avicennia marina* (Forssk.) Vierh. The dominant topographic element was recorded for each site. The salt marsh topographic classes were: edge of pool or depression; lower marsh surface or upper marsh surface (see [10]). The relative relief of marsh surfaces was on average between 0.2 and 0.5 metres above the low points (*i.e.*, after tidal ebb) (Dale, unpublished data [17]). The mangrove topographic classes include fringing forest; saltmarsh-mangrove interface (with locally variable levels) and hummocks [4]. The saltmarsh-mangrove interface class was an area of transition between saltmarsh and mangroves. Hummocks had a relative relief of 0.2 to 0.6 m (*i.e.*, 0.1 to 0.2 m above standing water that was 0.1 to 0.4 m deep after an ebb tide).

### 2.2. Data Collection

At each site, 15 sub-samples were collected, each of 15 cc of substrate. The sub-samples were then pooled and the resultant samples processed according to the sieving and floatation method, as described in [18,19]. Sieving (using 150 and 300 µm mesh sizes) and flotation is one of the simplest ways to extract eggshells from substrate samples and is suitable for extracting eggshells from a range of Aedes and Verallina species (e.g., see [4]) where the eggshell size is in the 150–300 µm range. The extraction method involves subsampling. Although this does not recover all the eggshells compared to complete examination, little information appears to be lost. Indeed simply recording the presence of eggshells may be a sufficient indication of mosquito larval habitats for operational purposes [20]. There were 233 samples from mangrove sites and 80 from saltmarsh sites.

### 2.3. Data Analysis

Data were log transformed ln(x + 1) to approximate a normal distribution and analysed using ANOVA. Where there were significant differences identified in the ANOVA, extended Student’s *t*-tests were used to identify the specific differences. All analyses were conducted using the JMP statistical package (version 5.0.1.2) (SAS Inst. Inc. Cary, NC, USA) [21].

## 3. Results and Discussion

Although the statistical results are presented in terms of the log-transformed data we will present real data in the descriptions for ease of interpretation.

### 3.1. Oviposition in Saltmarsh and Mangrove Sites

There was no significant difference between eggshells in saltmarsh or mangrove systems (F = 3.37, df 1, 311, *p* > 0.06). The mean raw values exceeded the 0.05/cc eggshell concentration threshold referred to above. In mangroves the mean was 0.73/cc (SD 0.03) and 0.24/cc (SD 0.05) in saltmarsh.

### 3.2. Topographic Relationships between Oviposition Sites within Mangrove and Saltmarsh Sites

Topographic class was significant for eggshell distribution (F = 28.28, df 5, 367, *p* < 0.01). The extended Student’s *t*-test results are shown in Table 1 (*t* = 1.97, *p* = 0.05). The hummock unit in the mangrove sites had the highest concentration of eggshells and was significantly different from all the other classes but also was variable, as indicated by the large Standard deviation. The saltmarsh pool edge, upper marsh surface and saltmarsh-mangrove interface were not significantly different from each other and had eggshell concentrations above the 0.05/cc threshold. Fewest eggshells were in the lower marsh surface and fringing mangroves.

**Table 1:** Results of the extended *t*-test. Classes with similar letters are not significantly different (showing the raw data).

Environment	Topographic Class	*T*-test Class	Mean/cc	Standard Deviation
Mangrove	Hummock	A	1.38	4.03
Saltmarsh	Edge pool/depression	B	0.45	0.90
Saltmarsh	Upper marsh surface	BC	0.23	0.51
Mangrove	Saltmarsh-mangrove interface	BC	0.09	0.07
Saltmarsh	Lower marsh surface	C	0.04	0.08
Mangrove	Fringing forest	C	0.01	0.03

An eggshell density of greater than 0.05/cc indicates a likely problem habitat for mosquitoes [6]. In both the mangrove and saltmarsh sites reported here eggshell densities were very much higher than that at both the general level and at the topographic class level (except for the fringing mangrove forest).

Although there were no differences in eggshell density in mangroves and saltmarshes generally there were significant differences within the systems. The results showing high numbers of eggshells in hummock areas are consistent with other observations in south east Queensland [4]. The results are also consistent with Florida research which found high eggshell densities in hummocks and also on the sloping banks of pools in mangroves [8]. The sloping banks of saltmarsh pools have also been shown to have large eggshell densities [22]. These all have topographies in common that satisfy the habitat requirements for successful production of mosquitoes. The elevation is such that water levels are variable. This facilitates the wetting, drying, wetting sequences necessary for oviposition, egg conditioning and larval survival [5]. In the mangroves larvae can move or be moved into impounded pools between hummocks and, in saltmarshes, downslope from marsh surfaces and pool margins into the pools and depressions where water ponds after tidal flooding. The relatively large variation in eggshell density within the hummock landscape may indicate local habitat characteristics within that class that affect oviposition and may be explained by further research into, for example, detailed flooding patterns or micro-topographic variation.

The information from the eggshell analyses is relevant to mosquito management in both the short term and longer term. In the short term, strategies may be developed to reduce the overall mosquito problem. Although it is not feasible to aerially spray larvicides on what may be small patches within a system (e.g., saltmarsh surface and pool margins or mangrove hummocks), nevertheless, interfering with the underlying habitat characteristics that favour mosquito production by minimal on ground alterations of specific parts of the system may be able to reduce the overall problem to a point where spraying can be reduced to times of major outbreaks. There is some evidence for this in the saltmarsh runnelling project at Coomera Island in sub-tropical Queensland [23] and at a pilot project mangrove site in northern New South Wales where a single connection was dug to the tidal source with some internal linkages between and across hummocks. This reduced the need to spray larvicides to one treatment compared to six at nearby similar sites (Falkner personal communication [24]).

In the longer term the impacts of sea level rise will affect intertidal mosquito habitats. It has been shown in subtropical Queensland and elsewhere that mangroves are invading saltmarsh and the question is whether this might result in changes to mosquito production [25]. Based on the eggshell analysis there may be little difference between mangrove and saltmarsh overall. However if the mangrove hummock topography replaces the upper marsh surface (and relative elevations are similar for both hummocks and upper marsh surfaces) then mosquito production could increase significantly. This has implications for resourcing and implementing mosquito management. Mangroves are more difficult to treat than saltmarshes, as spraying may be impeded by canopy cover and, in some cases where flushing is poor, acid production may reduce the effectiveness of some larvicides and the associated reduction in dissolved oxygen [26] will reduce predatory fish access. Increasing tidal flushing by minor modification in strategic parts of the system can improve water quality (Knight unpublished data [27]), minimising the risk of less than optimal larvicide treatment outcomes and maximising fish access.

## 4. Conclusions

The eggshell research has enabled comparisons across systems that would be difficult to achieve with larval surveys, as larvae are ephemeral whereas eggshells are more stable. It has shown that mangroves and saltmarshes are equally important for mosquito production. It has also shown that, within the systems, particular topographic types are significantly more productive than others and this has implications for both current control methods and for future control, as sea level rise leads to changing distributions of mangroves and saltmarsh. A major issue is the management of mosquito populations in mangroves as these systems are likely to become more prevalent in coming decades and are generally more difficult to treat. It is suggested that some minor modification within the systems may help to offset the potential for increased mosquito production.
